# Chromatin Buffers Torsional Stress During Transcription

**DOI:** 10.1126/science.adv0134

**Published:** 2026-03-26

**Authors:** Jin Qian, Lucyna Lubkowska, Shuming Zhang, Chuang Tan, Yifeng Hong, Xiaomeng Jia, Robert M. Fulbright, James T. Inman, Taryn M. Kay, Joshua Jeong, Glenn Hauk, Deanna Gotte, James M. Berger, Mikhail Kashlev, Michelle D. Wang

**Affiliations:** 1 Department of Physics & LASSP, Cornell University; Ithaca, NY 14853, USA; 2 Howard Hughes Medical Institute, Cornell University; Ithaca, NY 14853, USA; 3 RNA Biology Laboratory, Center for Cancer Research; National Cancer Institute, Frederick, MD 21702, USA; 4 Department of Electrical and Computer Engineering, Cornell University; Ithaca, NY 14853, USA; 5 Biophysics Program, Cornell University; Ithaca, NY 14853, USA; 6 Department of Biophysics and Biophysical Chemistry, Johns Hopkins University School of Medicine; Baltimore, MD 21205, USA; 7 HIV Dynamics and Replication Program, Center for Cancer Research, National Cancer Institute; Frederick, MD 21702, USA

## Abstract

During eukaryotic transcription, Pol II must overcome nucleosome obstacles and, because of DNA’s helical structure, must also rotate relative to DNA, generating torsional stress. However, there is limited understanding of how Pol II transcribes through nucleosomes while supercoiling DNA. Here, we determined that Pol II generates a torque of 9 pN·nm alone and 13 pN·nm with TFIIS, making it a powerful rotary motor. Intriguingly, when Pol II encounters a nucleosome, passage becomes more efficient on a chromatin substrate than on a single-nucleosome substrate, demonstrating that chromatin significantly buffers torsional stress during transcription. Furthermore, topoisomerase supercoiling relaxation allows Pol II to transcribe through multiple nucleosomes. Our results reveal a novel role of chromatin beyond the more conventional view of it being just a roadblock to transcription.

## One-Sentence Summary

Chromatin buffers torsional stress generated by Pol II elongation, facilitating transcription through a nucleosome.

Because of the DNA helical nature, during transcription elongation, RNA polymerase (RNAP) and DNA must rotate relative to each other as RNAP translocates along the DNA. *In vivo*, RNAP rotation is thought to be restricted due to the viscous drag of large molecular machineries, such as spliceosomes, associated with the nascent RNA (1), thus, the DNA at the RNAP must rotate. This obligatory relative rotation, dictated by the helical nature of DNA (2, 3), imposes one turn for every 10.5 nt transcribed by RNAP. Consequently, RNAP tightly couples its translocation and rotation, making it concurrently a linear motor to generate force to translocate and a rotary motor to generate torque to rotate (4, 5). This torque measures the torsional stress that RNAP experiences while supercoiling DNA. These inherent motor properties are crucial for gene expression, enabling RNAP to overcome roadblocks and reconfigure DNA structures and topology (6, 7). Notably, the rotational motion leads to DNA supercoiling and torsional stress in the DNA, which, in turn, regulates transcription (6–14). It is important to note that while supercoiling refers to extra turns introduced into DNA, torque measures how difficult it is to introduce those turns, reflecting RNAP’s capacity to rotate the DNA while working against the resistance. Yet our understanding of how RNAP generates torque and works against torsional stress is limited. Previously, we found that *E. coli* RNAP is a powerful rotary motor capable of generating sufficient torque to melt DNA (13, 14). Whether the eukaryotic RNA polymerase II (Pol II) can do the same remains unknown.

Recent studies have highlighted the diverse critical roles of transcription-generated DNA torsional stress in cellular functions (15–19). Torsional stress on DNA is present across the genome and regulates gene expression, even in the presence of native topoisomerases (15, 20–27). The torsional stress generated from the transcription of one gene can also regulate the expression of other genes in the vicinity (7, 10, 28, 29). Moreover, DNA torsion accumulation during a head-on conflict of transcription and replication has been suggested to promote persistent replication stress and subsequent DNA damage (30–32). It is worth noting that because torsional stress in DNA can act over a distance, this consequence may occur well before any physical encounter of RNAP with the replisome (30, 33). Furthermore, transcription-generated torsional stress has also been found to significantly change DNA topology by regulating 3D genome knotting, folding, loop formation, and DNA structures (34, 35).

Importantly, eukaryotic RNAP Pol II must transcribe through nucleosomes; thus, transcription through chromatin under torsion represents a fundamental problem in biology. Despite the significance of this problem, little is known about how Pol II transcribes through chromatin while supercoiling DNA. Chromatin is commonly perceived as a roadblock to transcription elongation, but this perception must be revisited, especially in the context of transcription under torsion. Curiously, while DNA assumes a right-handed helical structure, each nucleosome in chromatin has DNA wrapping around the core histones in a left-handed fashion (36, 37). However, it is unclear how DNA chirality and nucleosome chirality work together to impact Pol II’s ability to transcribe through nucleosomes. The complexity of this problem poses significant challenges to experimental investigation and requires new methodologies and conceptualizations to gain mechanistic understanding. Previous structural, biochemical, and single-molecule studies focused on transcription through a single-nucleosome without consideration of transcription-generated torsional stress. In this work, we have enabled this investigation by developing several highly sophisticated single-molecule methods. Our study has uncovered significant and quantitative insights into a novel role of chromatin during transcription under torsion.

## Visualizing Pol II rotation of DNA

The fundamental cause of transcription-generated torsional stress is RNAP rotation of torsionally constrained DNA. Understanding this rotation requires a real-time method for visualizing the rotational motion when transcription occurs at a specified torsional stress level. Previously, the rotational motion for *E. coli* RNAP was detected/inferred via the rotation of a bead (38), the rotation of a DNA origami attached to the DNA (39), or RNAP-generated DNA supercoiling (13, 14). However, these methods either do not allow on-demand control of the torsional stress or do not directly track the rotation.

Here, we present an approach to directly track Pol II-generated rotation of DNA at high spatial and temporal resolution using an angular optical trap (AOT). A defining feature of the AOT is its trapping particle, a nanofabricated birefringent quartz cylinder (Fig. 1a; Fig. S1), which serves as a handle for simultaneous measurements of force, extension, torque, and rotation of DNA (40–44). The angular orientation of the quartz cylinder can be detected at an exceedingly high angular and temporal resolution (45–47) and thus can be used to visualize how Pol II induces DNA rotation.

To apply the AOT to Pol II, we torsionally constrained *S. cerevisiae* Pol II (Fig. S2) to the coverslip surface of a sample chamber and its downstream DNA to the bottom of the cylinder (Fig. 1a; Figs. S3 & S4; Tables S1 & S2). Pol II translocation leads to positive (+) torque buildup, which ultimately buckles the DNA to form a plectoneme (Fig. S5). Once the torque reached a desired value, the AOT rotates the cylinder to follow Pol II rotation of DNA, thus limiting further torque buildup and maintaining a constant torque. At this point, for each turn of Pol II rotation of the DNA (10.5 bp transcribed), the cylinder also rotates by a turn (Methods). Because the cylinder’s extraordinary axis angle can be tracked at exceedingly high spatial and temporal resolution (45–47), this method provides unprecedented resolution of the Pol II rotational motion.

The example trace in Fig. 1b shows Pol II rotation of DNA under 3.2 pN·nm resisting torque. This torque is relatively low compared with the magnitude of the torque required to melt DNA (42, 48), mimicking an *in vivo* condition where topoisomerases can almost keep up with transcription. As Pol II progresses along the DNA, it exhibits steady rotational motion (1–2 turns/s), interspersed by pauses. This rotational motion, also visualized in Movie S1, corresponds to an overall rate of 10–20 nt/s elongation rate, comparable to those determined when RNAP rotation was not topologically constrained in previous *in vitro* experiments (49–55). This indicates that a torque of 3.2 pN·nm does not significantly slow down Pol II elongation. These Pol II rotational behaviors are also reminiscent of the translocational motions of RNAP from previous studies (49–55), reflecting the translocation-rotation coupling of RNAP. The direct visualization of the Pol II rotational motion highlights the inherent nature of rotational motion during transcription and invites the investigation of the inevitable consequences of transcription-generated rotation.

## Pol II is a powerful rotary motor

*In vivo*, Pol II-generated DNA supercoiling cannot be fully dissipated by rotation of the DNA ends, which are often associated with cellular structures, domains, or factories (1). Consequently, transcription accumulates positive (+) supercoiling and torsional stress in front of Pol II and negative (−) supercoiling and torsional stress behind, leading to the well-known “twin-supercoiled-domain” model of transcription (27, 56). Both the (+) torsional stress in front of Pol II and the (−) torsional stress behind it hinder transcription. These torsional buildups can occur even in the presence of topoisomerases (15, 20–27), suggesting that topoisomerases cannot always keep up with transcription. Especially when there is a transient shortage of topoisomerases near an active gene, torsional stress in the DNA can build up, leading to Pol II stalling. The torque at which Pol II stalls determines Pol II’s torsional capacity to work against torsional stress and modulate the DNA topology. However, it is unknown how much torque Pol II can generate before stalling.

To measure Pol II’s stall torque, we adapted a method we previously used for stall torque measurements of *E. coli* RNAP using an AOT (13, 14). Instead of rotating the cylinder as in Fig. 1a, we restricted the cylinder rotation, allowing Pol II to build up torsional stress on the DNA and eventually stall (Fig. 1c). During this process, we monitored how Pol II moves along the DNA and generates torsional stress in real-time (Fig. 1d). We investigated two stalling configurations: Pol II accumulation of (−) supercoiling behind or (+) supercoiling in the front and found that the stall torque magnitudes are similar in these two configurations despite being opposite in rotational sense: −9.0 ± 1.4 pN·nm (mean ± SD) and +8.5 ± 1.9 pN·nm, respectively. For comparison, the DNA melting torque is around −10 pN·nm (42, 48), suggesting that Pol II is a torsional motor almost sufficiently powerful to melt DNA.

## TFIIS enhances Pol II’s torsional capacity

Upon stalling under torsion, we observed that Pol II often reverses its movement and moves backward by up to 100 bp or more while relaxing the transcription-generated torsional stress (Fig. 1d; Fig. S6). This reverse motion is consistent with Pol II backtracking, during which Pol II reversely translocates along DNA, threading its 3’ transcribed RNA through the secondary channel and rendering Pol II inactive (57–62). This observation shows that backtracking is the primary cause for stalling under torsion, indicating that anti-backtracking transcription elongation factors, such as TFIIS (58, 63–65), may facilitate Pol II transcription under torsion by stimulating the cleavage activity of Pol II and rescuing the backtracked complexes in a way similar to the effect of GreB on *E. coli* RNAP (14).

Investigating whether TFIIS can facilitate Pol II transcription under torsion and, more broadly, how Pol II works against obstacles requires extensive exploration of broad experimental conditions, which is challenging when measurements must be conducted sequentially on the AOT. The scope of this investigation motivated us to develop an alternative method using magnetic tweezers (MT), which enable parallel measurements of multiple molecules while resolving the dynamics of each molecule (11, 12, 35, 66–70). Although the MT instrument cannot directly measure torque, the torque on the DNA substrate may be obtained through the measured DNA extension and force using the DNA torsional properties established from the AOT (Fig. S5; Table S2).

We first used the MT assay to determine the stall torque of Pol II in the presence of TFIIS. In this experiment, we anchored Pol II to a magnetic bead and torsionally constrained the downstream DNA to the coverslip surface of a sample chamber (Fig. 2a). Previously, we showed that *E. coli* RNAP stall torque may be obtained via a torque-jump method using the AOT (13). We adapted this method for Pol II stall torque measurements on the MT. We first verified that Pol II was active under an assisting torque by allowing it to transcribe under (−) supercoiling (Methods). Continued transcription converts the (−) supercoiling to (+) supercoiling and buckles the DNA to form a (+) buckled plectoneme at +6 pN·nm torque. We then jumped the torque to a higher value, for example, +10 pN·nm (Fig. 2b), and determined whether Pol II could continue moving forward (Methods). In the example trace without TFIIS (Fig. 2b), Pol II stalls and extensively backtracks, with the backtracking distance increasing with time, indicating +10 pN·nm is significant resistance to forward movement. In contrast, in the presence of TFIIS (Fig. 2b), Pol II still backtracks. However, after backtracking, Pol II resumes forward translocation, consistent with TFIIS rescuing the backtracked elongation complex. Thus, Pol II continues to move forward despite the +10 pN·nm resistant torque.

*In vivo*, the torsional stress in DNA is regulated by topoisomerase activity: high torsional stress when topoisomerase activity is low, and low torsional stress when topoisomerase activity is high. Our torque-jump experiment mimics this process by examining Pol II activity at different torques. Fig. 2c shows the mean trajectories of Pol II. Under low torque values (such as 0 and +6 pN·nm), Pol II moves forward steadily, showing minimal differences with and without TFIIS. However, when the torque increases to +10 pN·nm, Pol II without TFIIS cannot sustain steady forward movement and backtracks extensively; in contrast, Pol II with TFIIS can still move forward. We examined the pause-free velocity, which reflects primarily the behavior of transcription elongation along the main reaction pathway (71, 72). The resulting torque-velocity relation, akin to the force-velocity relation and characteristic of the chemo-mechanical property of a motor protein (13, 72), shows that Pol II pause-free velocity decreases with an increase in the resisting torque (Fig. 2d). Although TFIIS dramatically increases the overall ability of Pol II to move forward against torsional stress (Fig. 2c), TFIIS does not increase the pause-free velocity (Fig. 2d), consistent with TFIIS acting only on the backtracked elongation complex of the branched pathway. Interestingly, the measured torque-velocity relation shows a good agreement with that expected of *E. coli* RNAP (71, 72) (Fig. S7), suggesting that Pol II may have energetic and kinetic properties in the main reaction pathway similar to those of *E. coli* RNAP.

To determine the stall torque of Pol II, we measured the fraction of Pol II molecules remaining active at different torques (Fig. 2e). We define stall torque as the torque when 50% of the molecules remains active (13). This analysis shows that Pol II alone can generate +8.6 ± 0.3 pN·nm torque, consistent with values measured using the AOT within the measurement uncertainty. The presence of TFIIS increases Pol II stall torque significantly to +12.8 ± 0.4 pN·nm, representing a 49% enhancement. Thus, TFIIS enables Pol II to work more effectively against torsional stress.

As a comparison, *E. coli* RNAP alone generates ~ +11 pN·nm torque (13). So, Pol II generates a torque comparable to *E. coli* RNAP, though slightly smaller. In addition, *E. coli* RNAP’s torsional generation capacity is also enhanced by the anti-backtracking factor GreB to +18.5 pN·nm torque (14). Thus, the torsional capacity enhancement by anti-backtracking transcription elongation factors may be general to all transcription machineries. These factors may rescue backtracked complexes, and subsequent resumption of transcription provides a renewed opportunity for RNAP to work against a greater torque (14). Overall, the basal machinery of eukaryotic transcription is a powerful rotary motor whose torsional capacity can be regulated by transcription factors. *In vivo*, many other factors may potentially modulate the torsional capacity of Pol II to facilitate its passage through obstacles such as nucleosomes (73).

## Transcription through a nucleosome under torsion

In recent decades, through extensive *in vitro* and structural studies, we have gained significant mechanistic insights into the nature of the nucleosome barrier to transcription (58, 74–82). These studies have focused primarily on transcription through a single nucleosome under no torsional stress. In the cell, Pol II must transcribe through nucleosomes in chromatin while supercoiling DNA. Thus, Pol II simultaneously experiences a physical blockage from the encountered nucleosome and resistance from supercoiling buildup in the chromatin. How chromatin impacts the ability of Pol II to transcribe through nucleosomes under torsion has remained obscure due to the complexity of this problem and a lack of experimental approaches for investigation.

To tackle this problem, we developed a real-time, magnetic tweezers-based assay to track the progression of Pol II as it transcribes through nucleosomes in a chromatin fiber (Fig. 3a,b). The experimental configuration is similar to that of Fig. 2a, except that the downstream substrate contains either a single nucleosome or a chromatin fiber containing about 50 nucleosomes. To facilitate the comparison, the nucleosome-array template is identical to the single-nucleosome template in the number of base pairs, the transcription start site (TSS) location, and the sequence leading to and through the first nucleosome. Control experiments show that nucleosomes remain stable when subjected to a torque comparable to that Pol II might generate during transcription on a nucleosome on these templates (Fig. S8).

Prior to measurements, the measured DNA extension is substantially smaller for the nucleosome-array template due to the nucleosome compaction of DNA as expected (67, 68, 83, 84) (Fig. 3c). Upon introducing NTPs to the sample chamber, the magnetic tweezers are used to rapidly unwind each tether by 18 turns to (−) supercoil the DNA, facilitating Pol II’s escape into active elongation. Subsequent transcription converts the (−) supercoiling to (+) supercoiling. With continued transcription, Pol II introduces more (+) supercoils to DNA and eventually encounters a nucleosome. We use the real-time DNA extension to monitor Pol II position along the DNA during this process (Fig. S9; Methods).

In the representative traces (Fig. 3c), since Pol II position is indicated by its active site on the DNA template, the front edge of Pol II that encounters the nucleosome is about 20 bp downstream of the active site (85, 86). We found that Pol II pauses when encountering the first nucleosome at about 403 bp from the TSS on the single-nucleosome template or the nucleosome-array template. The presence of TFIIS significantly increases the ability of Pol II to move through the first nucleosome on either template. We examined the dwell time of Pol II when encountering a nucleosome in the presence of TFIIS (Fig. 3d). On the single-nucleosome template, Pol II pauses strongly before reaching the dyad of the nucleosome, with a pausing pattern indicative of periodicity (Fig. 3d left panel). Fourier transformation of this signal reveals an ~ 11-bp periodicity (Fig. S10), consistent with Pol II pausing at the strong histone-DNA interactions in a nucleosome, which occur whenever the DNA minor groove faces the histone core octamer surface (36, 87). This exceptional resolution results from the detection resolution enhancement of buckled DNA: each 10.5 bp of Pol II movement leads to a ~ 50 nm reduction to the DNA extension. The nucleosome-array template does not allow this resolution enhancement because the extension is about ten times less sensitive to Pol II movement (Fig. S11). Nonetheless, Pol II dwelling around the dyad is still detected (Fig. 3d right panel). For both templates, the pausing patterns bear resemblance to those of transcription through a nucleosome previously observed *in vitro* (76, 88, 89) or through nucleosomes in the gene body *in vivo* (90–92), with pausing occurring primarily before and around the dyad. These observations demonstrate that we have established a method to investigate how Pol II transcribes through nucleosomes under torsion.

## Chromatin buffers torsional stress to facilitate transcription

Intriguingly, the representative traces in Fig. 3c are suggestive that Pol II transcribes through a nucleosome more efficiently on the chromatin template than on the single-nucleosome template. To rigorously evaluate this observation, we pooled many traces and plotted the mean Pol II trajectory at their nucleosome encounter (Fig. 4a; Fig. S12; Methods). Indeed, Pol II transcription is more efficient on the chromatin template. In the absence of TFIIS, Pol II stalls extensively when it first encounters the nucleosome on the single-nucleosome template. In contrast, Pol II can significantly invade the nucleosome on the chromatin template. The presence of TFIIS greatly facilitates Pol II passage through a nucleosome on both templates, but transcription remains more efficient on the chromatin template.

It is important to note that for both templates, which are *identical* in length, Pol II transcribes the *same* DNA sequence and generates the *same* number of supercoils when encountering the nucleosome (Fig. 3a). Yet, the nucleosome-passage rates significantly differ (Fig. 4b). The only difference between the two configurations is the substrate downstream of the nucleosome that Pol II first encounters: naked DNA for the single-nucleosome template and chromatin fiber for the chromatin template. Our previous studies show that the torsional stiffness of chromatin fiber is about 8.5 times softer than that of naked DNA (67). The torsional stiffness reduction ultimately results from the left-handed chirality of a nucleosome with DNA wrapping around a histone octamer in a left-handed configuration, constraining (−) supercoiling within a nucleosome (67, 93, 94), with the entry and exit DNA segments being able to adopt different states. Under no torsion, these segments are more prone to adopt a (−) crossed state. The presence of (+) torsion converts these segments into a (+) crossed state (68, 95), limiting twist buildup without histone dissociation (67) (Fig. S8). Thus, the torsional mechanical properties of chromatin allow effective buffering of transcription-generated torsional stress on the chromatin template, facilitating transcription through the nucleosome. In contrast, the naked DNA of the single-nucleosome template cannot effectively buffer transcription-generated torsional stress, making torsional stress a significant obstacle to transcription. In a way, this is akin to Pol II twisting substrates of different physical properties: while it is easier to twist chromatin by adding some turns (imagine twisting a rope), it is considerably harder to twist naked DNA by adding the same number of turns (imagine twisting a metal rod) (5).

Consistent with this chromatin torsional mechanical explanation, our measured nucleosome-passage rate is greater by ~ 5.5-fold on the chromatin template than on the single-nucleosome template. Using the torsional mechanical properties of chromatin (67), we estimate that Pol II experiences only 1.6 pN·nm resisting torque when encountering a nucleosome on the chromatin template but 8.5 pN·nm resisting torque when encountering a nucleosome on the single-nucleosome template (Fig. 4b) (Methods; Fig. S11). To further evaluate this possibility, we also examined the nucleosome-passage rate on a single-nucleosome template that is not torsionally constrained so that torsional stress cannot accumulate on this template (0 pN·nm) (Fig. 4b; Figs. S12 & S13). Indeed, the nucleosome-passage rate on this template is significantly increased and even greater than that on the chromatin-fiber template under torsional stress (Fig. 4b), further demonstrating how torsional stress can impact the nucleosome-passage rate.

Collectively, our findings demonstrate the effectiveness of chromatin in buffering torsional stress during transcription. This buffering results from the left-handed nucleosome chirality and does not require the disassembly of nucleosomes downstream of Pol II (67) (Fig. S8). Although chromatin has been traditionally viewed as a roadblock to transcription, our results show that by buffering torsional stress, chromatin actually facilitates transcription, revealing a crucial role of chromatin in regulating transcription. Importantly, although DNA supercoiling and torsional stress are distinct physical quantities, they are often conflated in the literature. Our work shows how torsional stress can significantly impact transcription even under the *same* degree of supercoiling. Torsional stress (reflected by torque), not DNA supercoiling, dictates the difficulty of Pol II in twisting DNA. The distinction between DNA supercoiling and torsional stress is critical but historically overlooked. Our data help highlight this difference.

## Torsional stress modulates nucleosome passage

Even though chromatin is an effective torsional buffer, continuous transcription eventually generates enough torsional stress to exceed the buffering capacity of chromatin, limiting Pol II’s ability to transcribe through multiple nucleosomes. Thus, timely torsional relaxation by topoisomerases is crucial to ensure Pol II progression (15, 20–27). Since topoisomerases relax DNA supercoiling, they should reduce torsion in chromatin and facilitate the passage of Pol II through nucleosomes.

To investigate how topoisomerase relaxation facilitates transcription through chromatin, we conducted transcription experiments in the presence of human topoisomerase I (topo I) or yeast topoisomerase II (topo II) (Fig. 5a) using a configuration similar to that shown in Fig. 3a. Our control experiments show that both topo I and topo II can effectively relax torsional stress in a chromatin substrate (Fig. S14) (67, 68). However, the Pol II location on DNA can no longer be tracked in real-time by the DNA extension, because the DNA extension can be altered by topoisomerase relaxation or Pol II translocation and thus does not uniquely inform the Pol II location on the template. Instead, we assess the Pol II location using the extension-turns relation of the chromatin substrate downstream of Pol II. Previously, we have characterized how the width and height of the extension-turns relation of chromatin depend on the number of nucleosomes (67, 68). We now use this characterization to estimate the number of nucleosomes on the remaining chromatin between Pol II and the coverslip, which provides an estimate of the location of Pol II on the template (Methods). As Pol II progresses through nucleosomes, the chromatin array ahead of Pol II shortens, leading to a reduced width and height of the extension-turns relation (Fig. 5b). These features provide an estimation for the number of nucleosomes transcribed.

Using this method, we monitored how Pol II transcribes through chromatin as a function of time (Fig. 5c). In the absence of any topoisomerases, Pol II can transcribe through one nucleosome, consistent with chromatin effectively buffering torsional stress during transcription. However, after the passage of this nucleosome, Pol II significantly slows down while transcribing through the second nucleosome, consistent with torsional stress approaching the buffering capacity of chromatin (Fig. S11) and suggesting that further transcription requires torsional relaxation by topoisomerases. Indeed, when either topo I or topo II is present, Pol II can continue to transcribe through multiple nucleosomes with less indication of being significantly slowed down over time. Interestingly, topo I is more effective in allowing more efficient nucleosome bypass. Thus, the presence of topoisomerases ensures more processive transcription.

*In vivo*, Pol II may also encounter other motor proteins that share the same DNA substrate, and Pol II progression is modulated by torsional stress generated by those processes (Fig. S15). For example, when one Pol II trails another Pol II moving co-directionally, the leading Pol II generates (−) supercoiling behind that annihilates the (+) supercoiling in front of the trailing Pol II, reducing the torsional stress in front of the trailing Pol II. Similarly, when a Pol II encounters another Pol II or a replisome moving head-on, (+) supercoiling accumulates between the two motors, increasing the torsional stress.

To investigate how additional torsional stress impacts transcription, we allowed Pol II to transcribe on the chromatin template while either mechanically unwinding the DNA to reduce torsional stress or overwinding the DNA to increase torsional stress, mimicking the torsional action of other motor proteins. In this experiment, we rotated the magnet at a constant rate while monitoring the progression of Pol II through a nucleosome (Fig. 5d). As expected, unwinding the chromatin substrate facilitates Pol II progression through a nucleosome while overwinding the chromatin substrate hinders its progress (Fig. 5d,e). Therefore, the ability of Pol II to bypass a nucleosome is modulated by the level of the torsional stress.

Taken together, these results further highlight the role of torsional stress during transcription through nucleosomes and provide additional support for the torsional mechanical model of transcription. We found that topoisomerase relaxation of torsional stress can significantly facilitate transcription through chromatin, allowing Pol II to progress through multiple nucleosomes without significant hindrance from the torsional stress buildup. We also show that Pol II passage through a nucleosome can be modulated by additional torsional stress, which could be imposed by other motors on the same substrate. These results demonstrate how torsional stress can modulate transcription through nucleosomes.

## Discussion

Our findings show that torsional stress is a strong regulator of transcription through chromatin. We have provided unprecedented information on Pol II transcription dynamics through chromatin under torsion. This work shows a complex interplay of DNA supercoiling generated by Pol II and chromatin’s ability to buffer the resulting torsional stress. The direct comparison of transcription through a nucleosome on a single-nucleosome template and a chromatin template also calls attention to the crucial distinction between DNA supercoiling and torsional stress, which have often been conflated in the literature.

We visualized Pol II rotation of DNA, demonstrating the inevitable consequence of torsional stress associated with transcription (Fig. 1a,b). Although RNAP has primarily been examined as a linear motor that generates force to translocate along the DNA (49, 50, 52, 54), this work on Pol II, together with previous studies of *E. coli* RNAP (13, 14), brings to the fore that RNAP is also a rotary motor that generates torques to rotate the DNA. We found that Pol II can generate a torque of about 9 pN·nm that nearly melts the DNA and becomes significantly more powerful in the presence of TFIIS to about 13 pN·nm (Fig. 1; Fig. 2). Thus, Pol II is a potent rotary motor with its torsional capacity modulated by transcription factors. While this work focuses on the basal transcription machinery with TFIIS, other factors (96–99) will likely further modulate the torsional capacity of Pol II *in vivo.*

Our data demonstrate that chromatin can effectively buffer (+) torsional stress generated ahead of transcription, allowing Pol II to progress more efficiently through a nucleosome, a feat unattainable if the downstream is naked DNA (Fig. 3; Fig.4). Although chromatin has been previously proposed to serve as a buffer of torsional stress (67, 95, 100, 101), our data represent the first experimental evidence to support this hypothesis in the context of transcription and demonstrate the role of chromatin beyond being just an obstacle. Importantly, this (+) torsional buffering results from the nucleosome chirality and does not require nucleosome disassembly (67, 68, 95) (Fig. S8). Thus, the left-handed nucleosome chirality coordinates with the right-handed DNA to buffer (+) torsional stress and facilitate transcription through nucleosomes. Interestingly, genomics studies show that nucleosomes are present within gene bodies at high occupancy, even for highly transcribed genes (102, 103), and deletion of factors that preserve nucleosome integrity on the chromatin could result in a down-regulation of genic transcription (104). Thus, chromatin may be a necessary substrate constituent to limit torsional stress accumulation during active transcription.

Previous *in vivo* studies also show that transcription-generated (+) torsional stress could promote destabilization of nucleosomes ahead of Pol II (22, 105), since each nucleosome traps about one (−) turn by the left-handed wrapping of DNA around a nucleosome (93, 94). We previously found that +19 pN·nm can facilitate the dissociation of the H2A/H2B dimers in a nucleosome (106), suggesting that the torque generated by Pol II in this work may not be sufficient to disassemble nucleosomes. Our control experiment shows that nucleosomes remain stable during the measurement time under a torque comparable to that generated by Pol II during transcription through a nucleosome (Fig. S8). Although Pol II-generated (+) torsion is insufficient to disassemble nucleosomes in our experiments, it could still help to destabilize the nucleosomes ahead of Pol II. *In vivo*, Pol II could generate greater (+) torsion by interacting with other elongation factors, enhancing its ability to destabilize nucleosomes. In addition, nucleosome remodelers and chaperones could also slide and destabilize the nucleosomes, along with the assistance of (+) torsion, to allow Pol II passage (104, 107–112).

We also demonstrate that topo I or topo II can significantly facilitate transcription through multiple nucleosomes (Fig. 5a–c). Interestingly, topo I is more effective than topo II in this role. This difference may reflect how each enzyme interacts with its substrate. While topo II relaxes DNA via a strand passage mechanism (113) and prefers binding to a DNA crossover (114, 115), topo I relaxes DNA by interacting with a single DNA segment (116). On a chromatin substrate, topo II can bind to a crossover formed by the juxtaposition of the entry and exit DNA segments of a nucleosome. However, (+) torsion at a nucleosome first displaces the entry and exit DNA segments before bringing them together into juxtaposition. This leads to fewer DNA crossings being available for topo II binding until significant (+) torsion buildup occurs, and thus a delayed action of topo II in assisting transcription through nucleosomes. This is consistent with our previous finding that topo II has a faster relaxation rate and is more processive on chromatin under high (+) torsion stress (68). In contrast, topo I may perform its function by simply binding to the linker DNA between nucleosomes without the need to capture a crossover, thus allowing for more rapid and processive supercoiling relaxation. In support of this interpretation, previous *in vivo* studies show that topo I is the primary enzyme for relaxing transcription-generated torsional stress (22, 105), and topo I, but not topo II, acts within gene bodies (22, 27). Furthermore, as the torsion increases with the level of transcription, both topo I and topo II enrichment levels also rise, suggesting that torsion might facilitate topoisomerase recruitment or retention (27). In addition to topoisomerases, other motors in the vicinity can further modulate torsional stress (18, 117). We further show that transcription through chromatin can be upregulated by (−) torsion or down-regulated by (+) torsion downstream of Pol II for the range of supercoiling introduced in Fig. 5f.

While the torque capacity of Pol II may facilitate transcription against torsional resistance, this property may also exacerbate conflicts with other motor proteins. For example, when Pol II encounters a replisome head-on, (+) torsional stress accumulates between Pol II and the replisome, which could occur well before Pol II physically encounters the replisome since torsional stress can act over a distance. Previous studies support a model in which (+) torsional stress accumulation is the culprit for replication stress, leading to replication fork stalling and disassembly (30–32). Although the replisome often ultimately wins the conflict (118–120), Pol II’s ability to move forward against strong torsional stress will allow a greater (+) torsional buildup between the two motors that leads to greater fork instability, which is detrimental to genome integrity.

Despite chromatin being traditionally viewed as a hindrance to fundamental DNA processes, our findings demonstrate a new role of chromatin in promoting transcription under torsion. Previously, we provided evidence that chromatin facilitates replication by partitioning supercoiling behind the replication fork, simplifying the topology for subsequent chromosome segregation (67). This work extends this idea in the context of transcription. These studies reveal the crucial role of chromatin’s torsional mechanical properties in fundamental DNA processes.

## Supplementary Material

Supplementary Materials

Qian et al Figure S1 movie

Materials and Methods

Figs. S1 to S15

Tables S1 and S2

Movie S1

## Figures and Tables

**Figure 1.**
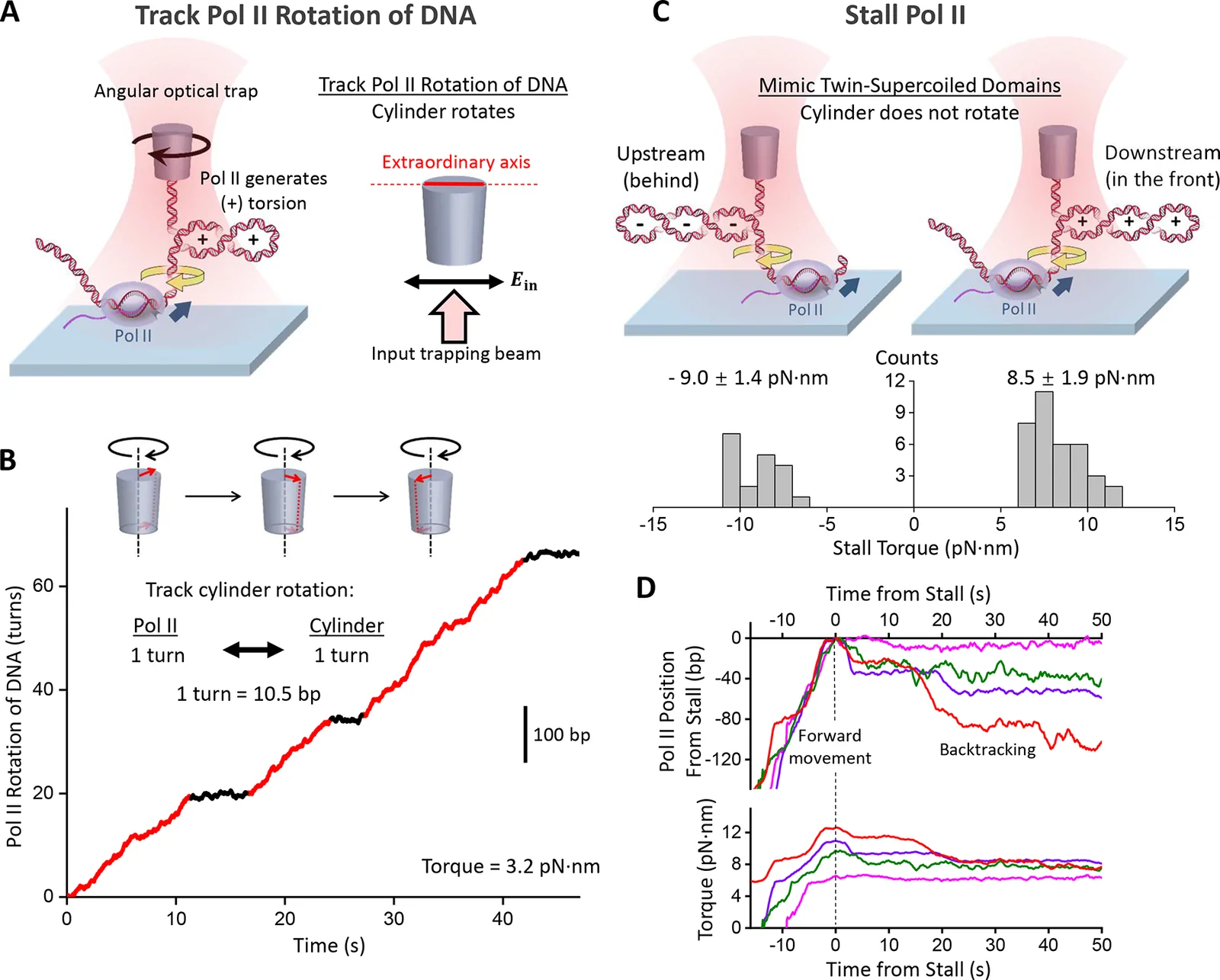
Visualizing Pol II rotation and measuring stalling under torsion. **a.** An experimental configuration to track Pol II rotation of DNA during transcription using an angular optical trap (AOT). Pol II is torsionally anchored on the surface of a coverslip, and its downstream DNA is torsionally attached to the bottom of a quartz cylinder, which is trapped by the AOT. Pol II elongation generates (+) supercoiling in the downstream DNA. The cylinder rotates to follow Pol II rotation to maintain a constant torque in the DNA. The right panel shows that the extraordinary axis of the cylinder tends to align with the linear polarization of the input trapping beam. The orientation of this axis is accurately detected and informs the angular orientation of the DNA attached to the cylinder. **b.** A representative real-time trajectory of Pol II rotation of DNA during transcription under a +3.2 pN∙nm resistance torque. The scale bar shows the conversion to base pair position of Pol II (approximating nucleotides transcribed). Regions of Pol II steady rotation are marked red, and regions of pausing are marked black. See Supplementary Movie 1 for the corresponding video. **c.** Pol II stall torque measurements using the AOT. The two cartoons illustrate the experimental configurations to stall Pol II against (−) torsional stress upstream (left) and (+) torsional stress downstream (right), mimicking the “twin-supercoiled-domain” model of transcription. The measured stall torque distributions are shown beneath the corresponding cartoon, with the mean and SD indicated. **d.** Representative traces of Pol II backtracking upon stalling under (+) torsional stress. Different colors represent different traces. Pol II forward translocates and stalls under increased (+) torque. Upon stalling, Pol II backtracks, as evidenced by the reverse motion.

**Figure 2.**
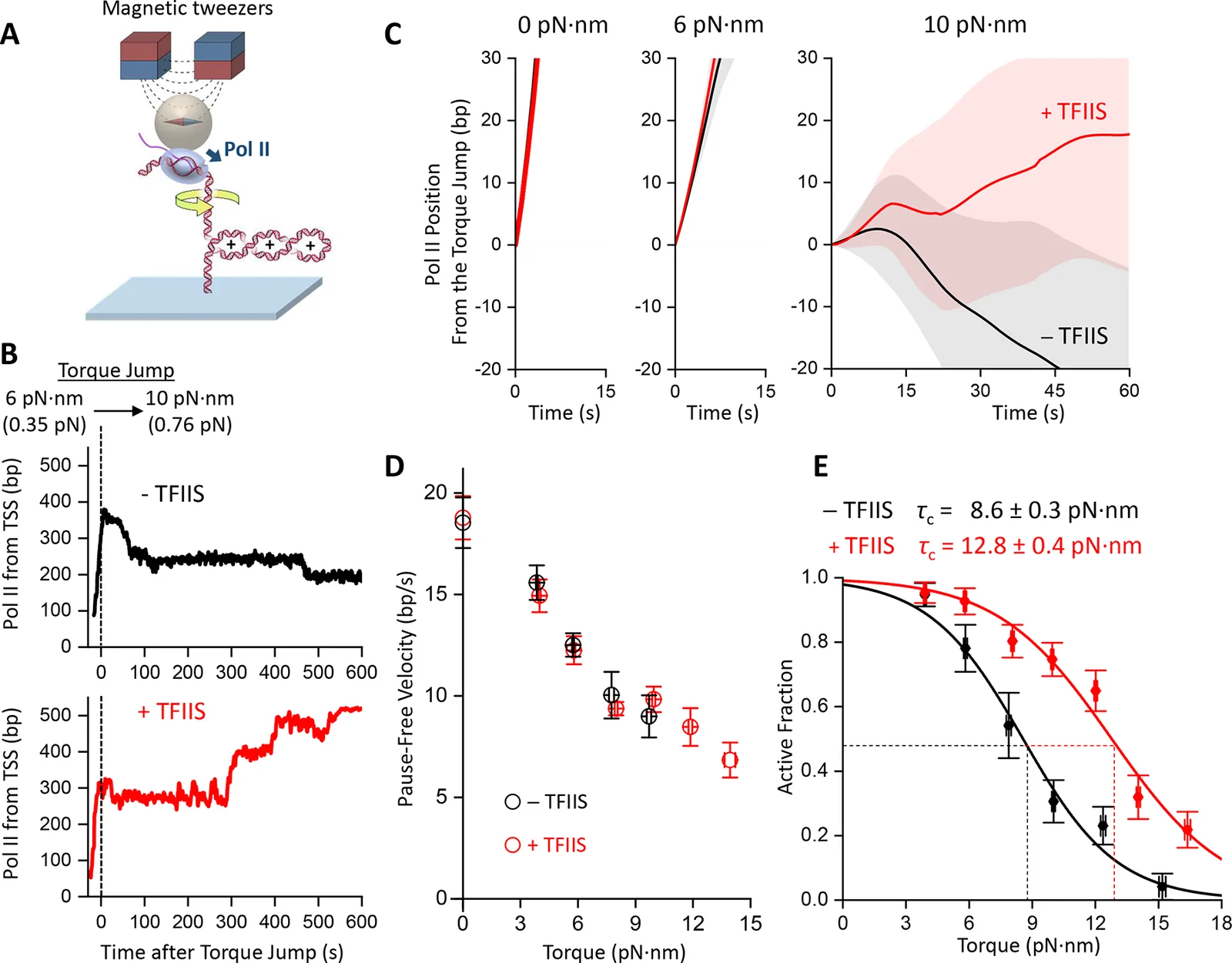
TFIIS up-regulates Pol II’s ability to transcribe under torsion. **a.** Experimental configuration to monitor transcription activity under torsion via magnetic tweezers (MT). Pol II is torsionally anchored to the surface of a magnetic bead, while its downstream DNA is torsionally anchored to the surface of a coverslip. The magnetic bead can be used to manipulate the torsional state of the DNA and the force on the DNA. **b.** Example traces of Pol II elongation during a torque-jump experiment. In these experiments, NTPs are introduced to the sample chamber before the measurements begin. During the NTP introduction, which typically takes about 20 s, each Pol II molecule may have moved a different distance from the transcription start site (TSS). Since the DNA is buckled during these measurements, the force on the DNA informs the torque in the DNA (Supplementary Fig. 5d). Pol II transcribes under a lower resisting torque, followed by a higher resisting torque. **c.** The mean trajectory of Pol II elongation under different torques. All traces were aligned at the start of the torque jump (*t* = 0), with the shaded region for each curve representing 30% of the standard deviation. Without TFIIS: *N* = 87, 72, and 42 traces for 0, 6, and 10 pN·nm, respectively. With TFIIS: *N* = 36, 72, and 61 traces for 0, 6, and 10 pN·nm, respectively. **d.** Torque-velocity relation. Pause-free velocities are shown without and with TFIIS. The error bars represent the SEM, with each data point collected from *N* = 27–87 traces. **e.** Active fraction of traces after the torque jump. The active fraction is the fraction of traces remaining active at a given torque. Each curve is fit with a decaying function (Methods) to obtain the critical torque *τ*_c_, at which 50% of traces are active. The fit values and uncertainties without and with TFIIS are also shown.

**Figure 3.**
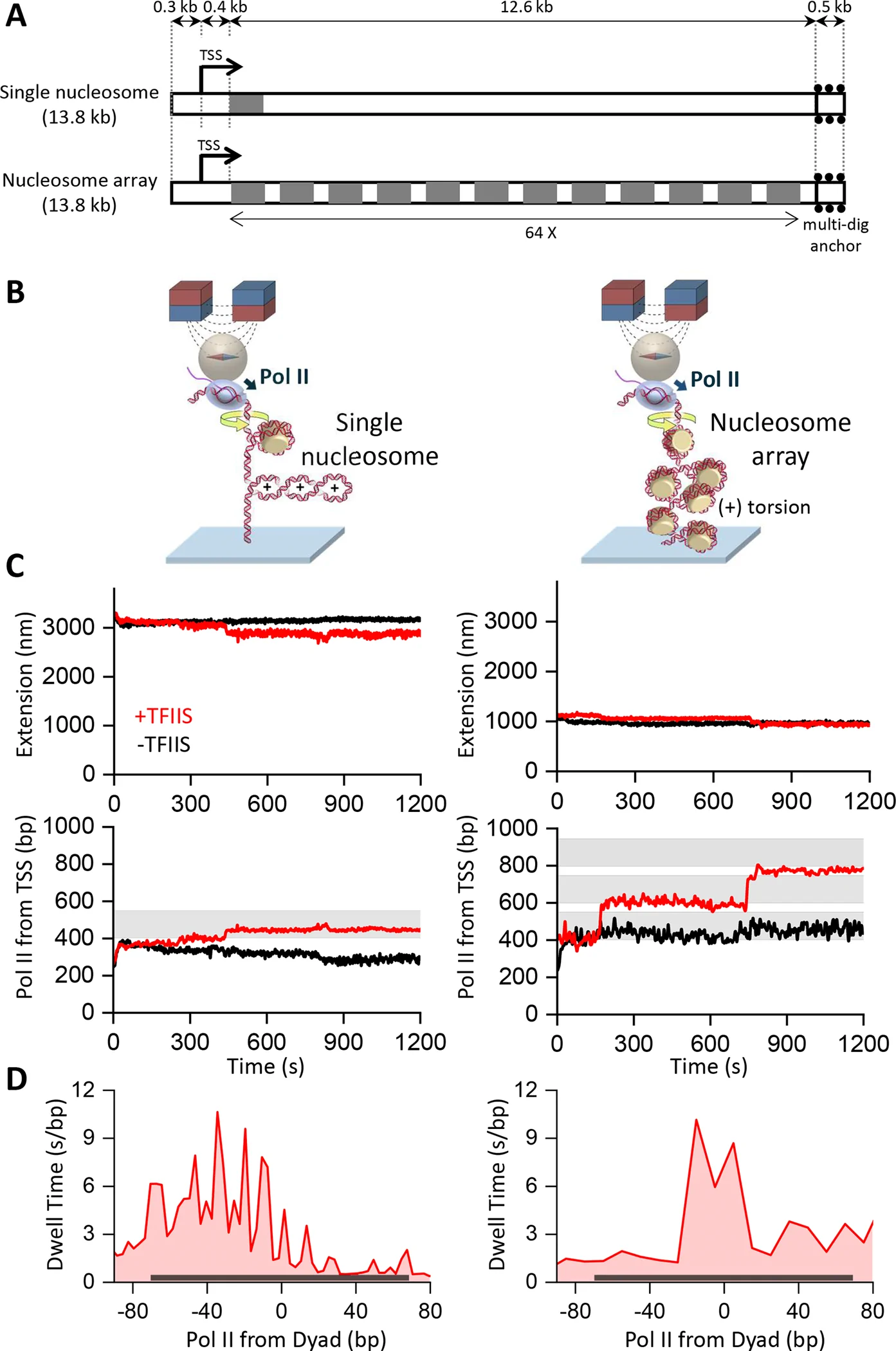
A real-time assay of tracking Pol II transcribing through nucleosomes under torsion via magnetic tweezers. **a**. The two DNA templates used for transcription through nucleosome experiments. Both templates have the same total length and the same sequence from the upstream end of the template (−313 bp from TSS) to the exit of the first nucleosome positioning element (NPE) (+550 bp from TSS). Each NPE is indicated as a grey box. The nucleosome array template contains 64 tandem NPE repeats with a repeat length of 197 bp. **b.** Experimental configurations. Pol II is torsionally anchored to the surface of a magnetic bead. The DNA downstream of Pol II contains either a single nucleosome (left) or a nucleosome array (right) and is torsionally anchored to the surface of a coverslip. **c.** Representative trajectories of Pol II transcribing through nucleosomes under torsion. The left panels show traces from the template containing a single nucleosome. The right panels show traces from the template containing a nucleosome array. The measured extension (top panel) is converted to the Pol II position from the TSS (bottom panel) for each trace (Methods). Each shaded region represents an expected nucleosome position. If a nucleosome dissociates from the DNA, there will be a sudden increase in the DNA extension of about 50 nm, which is not detected during these measurements. **d.** Pol II dwell time distribution at a nucleosome encounter. The dwell time at each position after Pol II encounters a nucleosome is calculated from Pol II trajectories of *N* = 58 traces (left panel) and *N* = 252 traces (right panel) using a “first-passage” method (Methods). The dark grey shaded region indicates the location of the NPE for the first nucleosome encountered.

**Figure 4.**
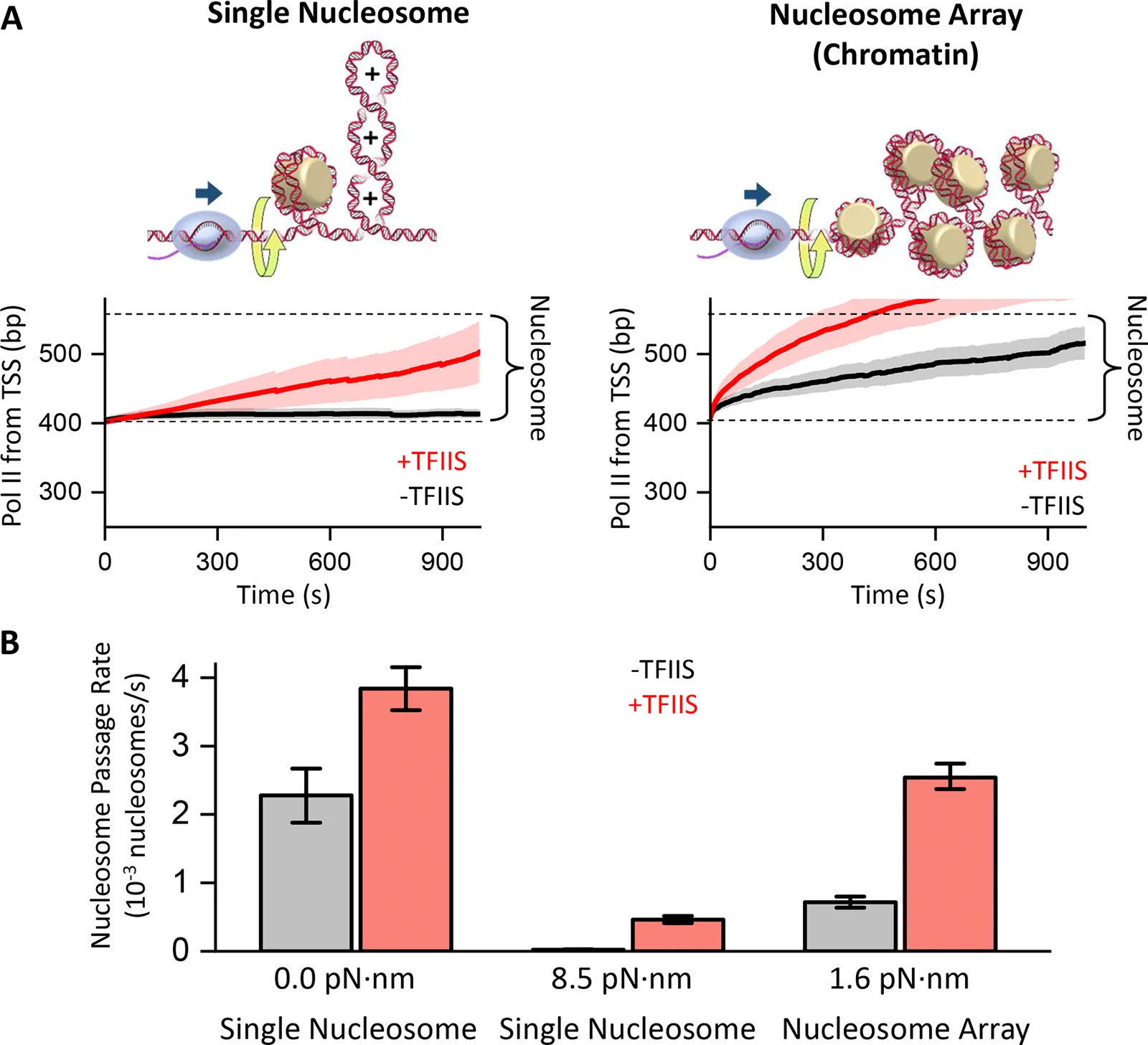
Chromatin buffers torsional stress to facilitate transcription. **a**. Mean trajectories of Pol II transcription through a nucleosome averaged over many traces. All traces are aligned in time when Pol II reaches the entry of the 1^st^ nucleosome encountered (*t* = 0), and the “first-passage” method is applied (Methods). The shaded regions represent 30% of the standard deviation. The left panel shows data from the single-nucleosome template (left; *N* = 86 traces for −TFIIS, *N* = 215 for +TFIIS). The right panel shows data from the first nucleosome on the chromatin template (right; *N* = 99 traces for −TFIIS; *N* = 302 traces for +TFIIS). **b.** Pol II nucleosome-passage rate. The mean passage rates under torsion were obtained from data shown in **a**, with the torque value being the torque experienced by Pol II when encountering the nucleosome dyad (Methods). The mean passage rates under zero-torsion of the single-nucleosome template were obtained from data shown in Fig. S13. The error bars represent the SEMs.

**Figure 5.**
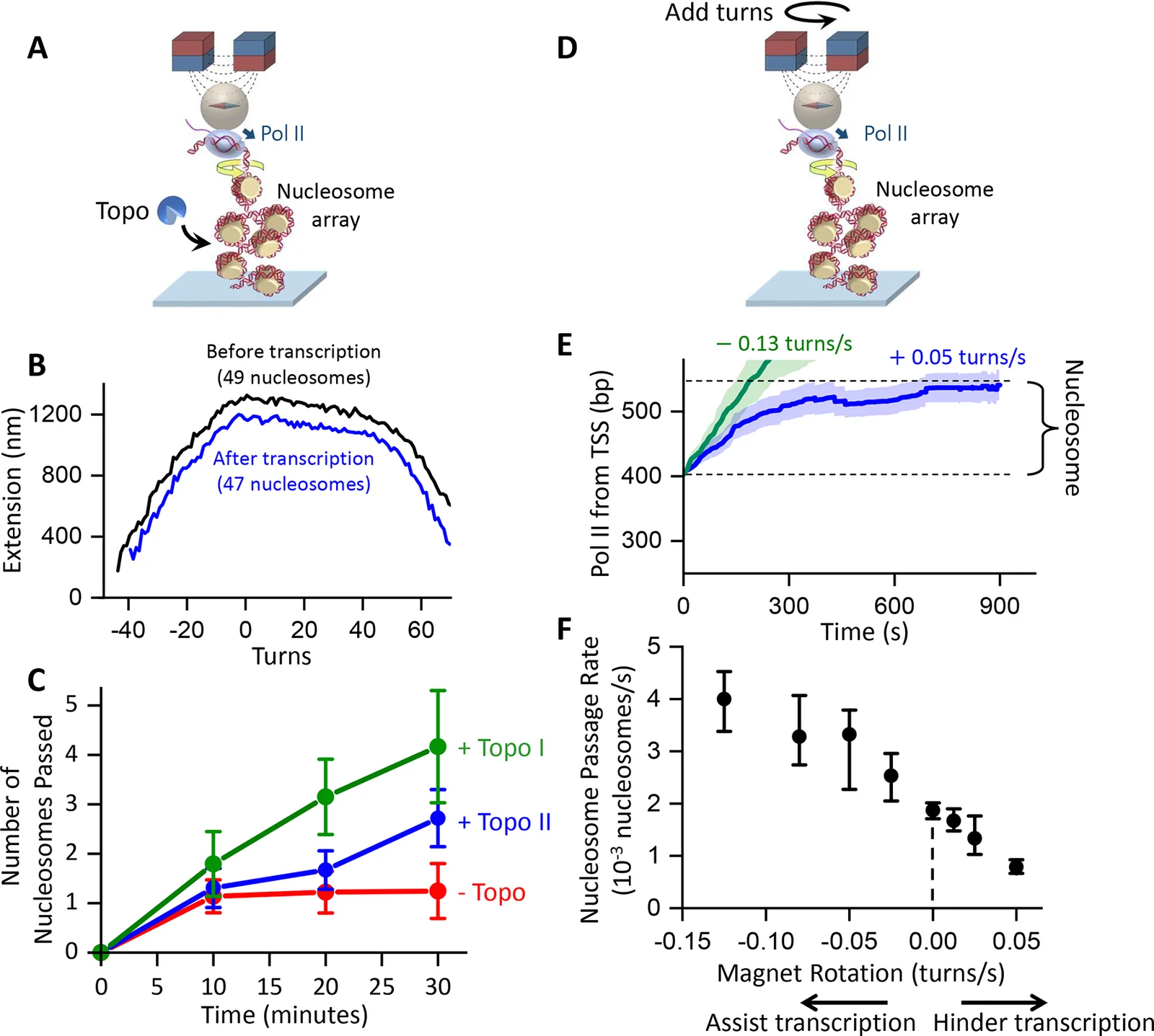
Torsional stress modulates nucleosome passage. **a.** Experimental configuration of transcription through chromatin in the presence of topo I or II. This configuration is identical to that used in Fig. 3b (right), except for having either topo I or topo II present in the assay. All experiments were carried out in the presence of TFIIS. **b.** Explanation of the method used to determine the number of nucleosomes passed. The extension-turns relation is determined by the number of nucleosomes and the DNA length between Pol II and the coverslip. Both the width and height of the extension-turns curve decrease after transcription. Shown are example traces where we detect 49 nucleosomes before transcription and 47 nucleosomes after transcription, indicating Pol II having passed 2 nucleosomes during transcription. For clarity of comparison, the curve after transcription is shifted laterally to re-center relative to the curve before transcription. **c**. The number of nucleosomes passed by Pol II during transcription versus time in the absence of topoisomerases or in the presence of either topo I or topo II. The error bars represent the SEM, with each data point collected from *N* = 14–33 traces. **d**. Experimental configuration to modulate torsional stress using the magnetic bead during transcription through chromatin. This configuration is similar to that used in Fig. 3b (right), except that the magnet bead is rotated at a constant rate. All experiments were carried out in the presence of TFIIS. **e**. Mean trajectories of Pol II transcription through a nucleosome at different magnet rotation rates. All traces were aligned when Pol II reached the entry of the 1^st^ nucleosome encountered (*t* = 0), with the shaded regions representing 30% of the standard deviation. Shown are examples of magnet rotation rates at +0.05 turns/s (hindering transcription) (*N* = 30 traces) and −0.13 turns/s (assisting transcription) (*N* = 49 traces). **f**. Pol II nucleosome-passage rates at different magnet rotation rates. The mean rates for the passage of the 1^st^ nucleosome encountered are shown, with the error bars representing the ± SEMs, with each data point collected from *N* = 25–65 traces.

## Data Availability

Data analysis routines used to process and generate plots are available in Github: https://github.com/WangLabCornell/Qian_et_al. All data are available in the main text or the supplementary materials. All materials are available upon request, which may require a material transfer agreement (MTA).
