# Supplementary material for: Chromatin Buffers Torsional Stress During Transcription
Source: Science. Author manuscript; Available in PMC 2026 Nov 14. (PMC13769021; doi:10.1126/science.adv0134)
Supplement: Supplementary file 1 [file NIHMS2215562-supplement-Supplementary_Materials.docx]

Supplementary Materials for

**Chromatin Buffers Torsional Stress During Transcription**

Jin Qian^1,2^, Lucyna Lubkowska^3^, Shuming Zhang^1,2^, Chuang Tan^1,2^, Yifeng Hong^4^, Xiaomeng Jia^1,2^, Robert M. Fulbright^1^, James T. Inman^1,2^, Taryn M. Kay^5^, Joshua Jeong^6^, Glenn Hauk^6^, Deanna Gotte^7^, James M. Berger^6^, Mikhail Kashlev^3^, and Michelle D. Wang^1,2,*^

^1^Department of Physics & LASSP, Cornell University; Ithaca, NY 14853, USA

^2^Howard Hughes Medical Institute, Cornell University; Ithaca, NY 14853, USA

^3^RNA Biology Laboratory, Center for Cancer Research, National Cancer Institute; Frederick, MD 21702, USA

^4^Department of Electrical and Computer Engineering, Cornell University; Ithaca, NY 14853, USA

^5^Biophysics Program, Cornell University; Ithaca, NY 14853, USA

^6^Department of Biophysics and Biophysical Chemistry, Johns Hopkins University School of Medicine; Baltimore, MD 21205, USA

^7^HIV Dynamics and Replication Program, Center for Cancer Research, National Cancer Institute; Frederick, MD 21702, USA

*Corresponding author. Email: [mwang@physics.cornell.edu](mailto:mwang@physics.cornell.edu)

Figure S1. Fabrication of anti-digoxigenin-coated quartz cylinders.

The nanofabrication process of the quartz cylinders is modified based on a previously established protocol (*40*, *43*, *67*). We first reacted the quartz wafer (Precision Micro Optics, PSQB-131332) with (3-aminopropyl) triethoxysilane (Sigma-Aldrich, 440140) solution. We then dry etched cylinders via a deep ultraviolet (DUV) lithography process. After mechanically cleaving the cylinders off from the wafer, we covalently coupled anti-digoxigenin (Roche, 11333089001) with a linker of glutaraldehyde (Sigma, G5882). These cylinders enable binding of multi-digoxigenin DNA anchors to its functionalized surface in a constrained configuration for DNA torsional studies.

Figure S2. SDS gel electrophoresis of purified proteins used in this work.

(**A**) SDS-PAGE analysis (4-12% Bis-Tris gel run with MES SDS buffer, Coomassie staining) of *Saccharomyces cerevisiae* Pol II. Purified Pol II before (lane 2) and after (lane 3) biotinylation, with Pol II subunits identified on the right. The molecular weights of the markers (lane 1) are indicated on the left.

(**B**) SDS-PAGE analysis (10% Bis-Tris gel run with MES SDS buffer, Coomassie staining) of *Saccharomyces cerevisiae* TFIIS. Purified TFIIS (lane 2) is resolved by gel electrophoresis. The molecular weights of the markers (lane 1) are indicated on the left.

(**C**) SDS-PAGE analysis (16% Tricine gel run with Tricine SDS buffer, Coomassie staining) of HeLa histones. Purified histones (lane 2) are resolved by gel electrophoresis, with each type of histones identified on the right. The molecular weights of the markers (lane 1) are indicated on the left.

(**D**) SDS-PAGE analysis (4-12% Tris-Glycine gel run with Tris-Glycine SDS buffer, Coomassie staining) of human topoisomerase I (topo I). Purified topo I (lane 2) is resolved by gel electrophoresis. The molecular weights of the markers (lane 1) are indicated on the left.

(**E**) SDS-PAGE analysis (4-12% Tris-Glycine gel run with Tris-Glycine SDS buffer, Coomassie staining) of *Saccharomyces cerevisiae* topoisomerase II (topo II). Purified topo II (lane 2) is resolved by gel electrophoresis. The molecular weights of the markers (lane 1) are indicated on the left (same as those shown in D).

Figure S3. Pol II elongation complex formation.

A “gapped” template for transcription elongation complex formation contains a 76 nt “gapped” region lacking the non-template strand (the template strand in black; non-template strand in blue; the initial bubble region red and underlined), flanked by dsDNA. A 14 nt RNA oligo (purple) with 9 nt complementary to the template strand is annealed to the “gapped” template. After Pol II binds to the RNA-DNA hybrid, a 76 nt non-template strand DNA oligo (green) with fully complementary sequence to the “gapped” region is added. The nicks at both ends of the “gapped” region are ligated.

Figure S4. The DNA templates used for the Pol II rotation and stalling experiments. Grey dash lines indicate ligation sites.

Figure S5. Torsional properties of naked DNA substrate. A 6.5 kb DNA (*68*) was used to obtain the torsional properties of naked DNA in the transcription buffer using the AOT.

(**A,B**). Extension-turns curves and torque-turns curves under different forces.

(**C**) Post-buckling or melting extension slope versus force. The values were obtained by performing a linear fit to the post-buckling or melting region of the extension-turns curve with error bars representing fitting uncertainties.

(**D**) Post-buckling or melting torque versus force. The values were obtained by performing a horizontal line fit to the post-buckling or melting region of the torque-turns curve with error bars representing fitting uncertainties.

Figure S6. Pol II backtracking distance after stalling under torsion using the AOT. The backtracking distance is determined after Pol II stalled for 60 s.

Figure S7. Comparison of Pol II and *E. coli* RNAP pause-free velocity versus torque relations.

The Pol II pause-free velocity versus torque relations with and without TFIIS are the same as those shown in Fig. 2D. For comparison, also shown are the measured *E. coli* RNAP data and predicted relation from a thermal ratchet model (*72*) after conversion of force $F$ to torque: $\tau=(10.5*0.338 nm)/(2\pi)F$. Such a conversion assumes that force and torque contribute equivalently to the work-related term in the free energy, i.e., $Fx=\tau\theta$, where $x$ is the RNAP displacement and $\theta$ is the rotational angle of RNAP relative to DNA. Given that RNAP tracks along the helical groove of DNA, which has a helical pitch of 10.5 bp and a contour length of 0.338 nm/bp, each base pair of RNAP movement corresponds to a linear displacement of *x =* 0.338 nm and a rotation *θ =* 2π/10.5. Accordingly, force can be converted to torque: $\tau=0.57 \mathrm{nm}F$.

Figure S8. Nucleosome array stability over time.

To assess the stability of the nucleosome arrays, we measured the number of nucleosomes in the array over time in a transcription buffer without transcription by subjecting a chromatin fiber to a torque value comparable to that Pol II might generate during transcription through a nucleosome under torsion (Fig. 4B).

(**A**) Measurements under no torque (*N* = 48 traces). Error bars represent the standard deviation of the mean.

(**B**) Measurements under (+) turns, corresponding to about 7.4 pN∙nm torque (*N* = 26 traces). Error bars represent the standard deviation of the mean.

No significant change in the number of nucleosomes over the experimental time window was observed. The results of this control experiment suggest that the nucleosomes remain stable without histone dissociation throughout the measurements of transcription through a nucleosome under torsion.

Figure S9. Pol II position determination.

(**A**) The position of Pol II on the DNA template during transcription is determined from the measured extension using the predicted extension-turns curve as a function of Pol II position.

(**B**) To demonstrate how this method works, we show examples of how the extension-turns curve changes as Pol II transcribes along the DNA template on a single-nucleosome template (left) or on a nucleosome array template (right). In each example, the initial extension-turns curve (black) is measured before Pol II transcription. Subsequent Pol II transcription decreases the DNA contour length and increases the DNA linking number density in the downstream DNA segment, resulting in scaling and a shift of the predicted extension-turns curve. The measured extension represents a single point on the predicted extension-turns curve and provides the position of Pol II. The final position of Pol II is further confirmed by the measured extension-turns curve at the end of an experiment.

Figure S10. Power spectral density of Pol II dwell time histogram when encountering the nucleosome on the single-nucleosome template.

The spectral density was obtained from the Fourier transform of data shown in Fig. 3D (left). It reveals a ~ 11 bp periodicity, which agrees well with the helical pitch of dsDNA.

**Figure S11.** **Torsional properties** **for the substrate downstream of the first nucleosome encountered under a force of 0.5 pN.**

(**A**) The single-nucleosome substrate. The downstream substrate is naked DNA.

(**B**) The nucleosome-array substrate.

The extension-turn curves and the torque-turn curves are reprinted from the reference (*67*), with permission from Elsevier. We use these curves to provide an intuitive explanation of the torque that Pol II must work against when encountering a nucleosome. Pol II elongation starts from the dashed line (−18 turns) (Methods). Pol II elongation adds (+) turns to the substrate and thus moves towards the right of each curve. The shaded regions represent nucleosomes that Pol II encounters. It is worth noting that the nucleosome positions indicated here are approximations of the analysis methods provided under the Methods.

Figure S12. Individual representative traces of Pol II through the 1^st^ nucleosome encountered.

All traces are aligned in time when Pol II reaches the entry of the 1st nucleosome encountered (*t* = 0), and the “first-passage” method is applied (Methods). The red and black curves are the mean trajectories with and without TFIIS, respectively. The individual traces are shown in various other colors.

(**A**) Pol II transcription through a single nucleosome under torsion. The red and black curves are the mean trajectories identical to those shown in Fig. 4A, left panel.

(**B**) Pol II transcription through the first nucleosome in a nucleosome array under torsion. The red and black curves are the mean trajectories identical to those shown in Fig. 4A, right panel.

(**C**) Pol II transcription through a single nucleosome under zero torsion. The red and black curves are the mean trajectories identical to those shown in fig. S13C.

Figure S13. Pol II transcription through a single nucleosome under zero torsion.

(**A**) Representative extension traces of Pol II transcribing through a single nucleosome under zero torsion.

(**B**) Pol II position converted from the extension data shown in a. The shaded region represents an expected nucleosome position. In this assay, it is difficult to resolve the Pol II position inside a nucleosome, so this position is estimated using a linear interpolation of the positions before and after Pol II encounters the nucleosome.

(**C**) Mean trajectories of Pol II transcription through a nucleosome. All traces were aligned when Pol II reached the entry of the nucleosome (*t* = 0). Both the “periodic boundary condition” and the “first-passage” methods are applied (Methods). The shaded regions represent 30% of the standard deviation (*N* = 234 traces for –TFIIS; *N* = 418 traces for +TFIIS).

Figure. S14. Topoisomerase relaxation of supercoiling in a chromatin substrate.

We employ the continuous winding assay to assess topoisomerase supercoiling relaxation of a chromatin substrate (*67*, *68*, *83*). Using the magnetic tweezers, turns are added to a chromatin substrate at about 1 turn/s under 0.5 pN force, and the DNA extension is measured over time. Because supercoiling is introduced into DNA at a constant rate, a decrease in the extension indicates an inability of the topoisomerase to keep up with the magnet rotation, and conversely, a lack of extension decrease indicates efficient topoisomerase supercoiling relaxation. In the absence of topoisomerases, the DNA extension decreases as more turns are added. In the presence of either 150 pM human topo I (*N* = 14 traces) or 100 pM yeast topo II (*N* = 17 traces), the DNA extension remains essentially unchanged, consistent with topo relaxation of the supercoiling introduced.

Figure. S15. Cartoon illustrating how processes downstream of a transcribing Pol II can modulate the torsion that Pol II encounters.

Pol II trailing behind another transcribing Pol II experiences reduced torsional stress. Pol II encountering another Pol II (or a replisome) head-on experiences increased torsional stress.

Table S1. Primer sequences.

| **Oligo Name** | **Sequence (5’ → 3’)** |
| --- | --- |
| RNA 14 | UUUUUAUCGAGAGG (Underlined sequence complementary to the gap) |
| ARP77 NTS | TCAGCCCTATAGGATACTTACAGCCATCGAGAGGGACAAGGCGAATACCCATCCCAATCGGCCTGCTGGTGACACC |
| ARP78 TS | GGTGTCACCAGCAGGCCGATTGGGATGGGTATTCGCCTTGTCCCTCTCGATGGCTGTAAGTATCCTATAGGGCTGA |
| Upstream NTS | GACCTAATTCGAGCTCGGTACCCGGGGATCC |
| Downstream NTS | TCAGCGGATCCTCTAGAGTCCTTCAGCGAT |
| Short TS | CTGAAGGACTCTAGAGGATCCGCTGAGGTGTCACCAGCAGGCCGATTGGGATGGGTATTCGCCTTGTCCCTCTCGATGGCTGTAAGTATCCTATAGGGCTGAGGATCCCCGGGTACCGAGCTCGAATTAG |
| 0.6 kb gapped PCR F | ACTACACCTAGTGCAGGGCGCGTACTATG |
| 0.6 kb gapped PCR R | TAATCCAAATCGATGGCCCGAAGAGTGGGTTTTAC |
| 0.6 kb gapped PCR R Nuc | ATTATACAGGGGCTGACCCGAAGAGTGGGTTTTAC |
| 4.6 kb center segment F | AATACTGTTCACCAACGATCTGGATCACG |
| 4.6 kb center segment R | TTTGAGCGTGGGTCTCGCGGTATCATT |
| 6.5 kb upstream segment F | GCCACCTGACGTCTAAGAAACCATTATTATCA |
| 6.5 kb upstream segment R | GCAGGTCCTTTCCGGCAATCAGG |
| 0.3 kb NPE segment F | TGCCAAAACAGCCCCTGTATCACTGCG |
| 0.3 kb NPE segment R | AAACCGGAAGACATGTGCTCGTTAGTTGGGTCGAC |
| 12.3 kb center segment F | TTAACTTGGTCTCTGCACGTTTTCAACAGTGATGAGG |
| 12.3 kb center segment R | ACCTTGCCATCGATTTGGGGTTGTAATAGTTTATCCG |

Table S2. List of physical parameters in single-molecule experiments.

| **Figures** | **Quantity** | **Source** |
| --- | --- | --- |
| Fig. 1B | Cylinder turns | Measured by AOT |
|  | Tether extension | Measured by AOT |
|  | Pol II rotation of DNA | Measured by cylinder turns, see Methods |
|  | Force | Measured by AOT |
|  | Torque | Reference the force measurement in fig. S5D, confirmed by AOT measurement |
| Fig. 1C-D, fig. S6 | Cylinder turns | Measured by AOT |
|  | Tether extension | Measured by AOT |
|  | Force | Measured by AOT |
|  | Torque | Reference the force measurement in fig. S5D, confirmed by AOT measurement |
|  | Pol II position | Derived from the measured tether extension, cylinder turns, and force, see Methods |
| Fig. 2B-E | Tether extension | Measured by MT |
|  | Magnet turns | Measured by MT |
|  | Force | Measured by MT |
|  | Torque | Reference the force measurement in fig. S5D |
|  | Pol II position | Derived from the measured tether extension, magnet turns, and force, see Methods |
|  | Pause-free velocity | Derived from the Pol II position and time plot |
|  | Active fraction | Derived from the Pol II position and time plot |
| Fig. 3C-D | Tether extension | Measured by MT |
|  | Magnet turns | Measured by MT |
|  | Force | Measured by MT |
|  | Pol II position | Derived from the measured tether extension, magnet turns, and force, see Methods |
| Fig. 4A | Tether extension | Measured by MT |
|  | Magnet turns | Measured by MT |
|  | Force | Measured by MT |
|  | Pol II position | Derived from the measured tether extension, magnet turns, and force, see Methods |
|  | Passage rate | Obtained from Fig. 4A & fig. S13C trajectories |
| Fig. 4B | Torque | Reference fig. S11 |
| Fig. 5B-C | Tether extension | Measured by MT |
|  | Magnet turns | Measured by MT |
|  | Force | Measured by MT |
|  | Pol II position | Derived from the measured tether extension, magnet turns, and force, see Methods |
|  | Number of nucleosomes | Derived from the measured tether extension versus turns relation, see Methods |
| Fig. 5E-F | Tether extension | Measured by MT |
|  | Magnet turns | Measured by MT |
|  | Magnet rotation speed | Measured by MT |
|  | Force | Measured by MT |
|  | Pol II position | Derived from the measured tether extension, magnet turns, and force, see Methods |
|  | Passage rate | Derived from the Pol II position versus time plot |
| fig. S5A-D | Cylinder turns | Measured by AOT |
|  | Tether extension | Measured by AOT |
|  | Force | Measured by AOT |
|  | Torque | Measured by AOT |
|  | Post buckling extension slope | Derived from the measured extension versus turns relation |
| fig. S8 | Tether extension | Measured by MT |
|  | Magnet turns | Measured by MT |
|  | Force | Measured by MT |
|  | Torque | Reference fig. S11 |
|  | Number of nucleosomes | Derived from the measured tether extension versus turns relation, see Methods |
| fig. S9 | Tether extension | Measured by MT |
|  | Magnet turns | Measured by MT |
|  | Force | Measured by MT |
| fig. S10 | Spectral density | Fourier transform analysis of Fig. 3D (left) |
| fig. S11 | Tether extension | Measured by MT |
|  | Magnet turns | Measured by MT |
|  | Force | Measured by MT |
|  | Nucleosome location | Derived from the known nucleosome position on the template and magnet turns |
|  | Torque vs turns curve | Reprinted from Le et al. (*67*) |
| fig. S12A-C | Tether extension | Measured by MT |
|  | Force | Measured by MT |
|  | Pol II position | Derived from the measured tether extension, magnet turns, and force, see Methods |
| fig. S13A-C | Tether extension | Measured by MT |
|  | Force | Measured by MT |
|  | Pol II position | Derived from the measured tether extension, magnet turns, and force, see Methods |
| fig. S14A-B | Tether extension | Measured by MT |
|  | Force | Measured by MT |

Movie S1. Real-time visualization of Pol II rotation of DNA.

This video animation shows an example trace of Pol II rotation of DNA under +3.2 pN∙nm resistance torque, as shown in Fig. 1B. The Pol II rotation of DNA is visualized via the rotation of the trapped nanofabricated quartz cylinder in the AOT, as the cylinder rotates to follow DNA rotation. The inset circle represents the top view of the cylinder with its angular orientation indicated by the red arrow. Only a portion of this trace (from 25 s to 40 s) is animated. As shown, Pol II continuously rotates the DNA (red regions of the curve) and is interrupted by brief pauses (black regions of the curve). The scale bar provides the conversion to distance transcribed.
